# Innovative Microneedle Patches for Psoriasis Treatment: A Dual Approach With Methotrexate‐Zinc and Difelikefalin for Enhanced Therapeutic Outcomes

**DOI:** 10.1002/smll.73958

**Published:** 2026-05-30

**Authors:** Xiaolong Xu, Chunhao Long, Yuze Zhou, Lujing Fei, Youyou Zhou, Min Qi

**Affiliations:** ^1^ Department of Burns and Plastic Surgery Shenzhen Hospital of Southern Medical University Southern Medical University Shenzhen China; ^2^ Department of Biochemistry School of Medicine Southern University of Science and Technology Shenzhen China; ^3^ Department of Plastic Surgery Xiangya Hospital Central South University Changsha China; ^4^ Department of Dermatology Shenzhen People's Hospital The Second Clinical Medical College Jinan University Shenzhen China

**Keywords:** difelikefalin, methotrexate‐zinc complex, microneedle patch, psoriasis, transdermal drug delivery

## Abstract

Psoriasis is a chronic inflammatory skin disease often accompanied by pruritus and a high risk of secondary infection. To address the limitations of current therapies, we developed a dual‐layer microneedle (MN) system that integrates methotrexate‐zinc (MTX‐Zn) for sustained anti‐inflammatory and antibacterial effects with difelikefalin (DFK) for rapid antipruritic action. MTX‐Zn was successfully synthesized through coordination chemistry and exhibited enhanced stability, preserved antiproliferative activity, and strong antibacterial effects. The GelMA/PVA‐based microneedles showed excellent mechanical strength, efficient skin penetration, and distinct spatial drug compartmentalization. In an IMQ‐induced psoriasis model, MTX‐Zn/DFK MNs markedly improved clinical symptoms, reduced epidermal hyperplasia, restored immune‐cell homeostasis, and decreased Th17/Th1‐mediated inflammation. DFK‐loaded MNs rapidly alleviated pruritus, reduced epidermal nerve‐fiber density, and inhibited Schwann cell migration. Transcriptomic analysis further revealed that DFK suppressed Schwann cell EMT pathways. Together, this dual‐functional microneedle platform provides a synergistic therapeutic strategy that combines rapid itch relief with long‐term anti‐psoriatic and antibacterial effects, representing a promising approach for improving psoriasis management.

## Graphical abstract

This study presents a novel dual‐layer microneedle platform for psoriasis treatment, integrating rapid itch relief with sustained immunomodulatory and antibacterial effects. By combining difelikefalin and methotrexate‐zinc, this system effectively targets pruritus, epidermal hyperplasia, immune dysregulation, and infection, offering a promising, minimally invasive therapeutic approach for enhanced psoriasis management.

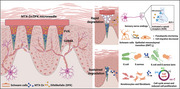

## 1 Introduction

Psoriasis is a chronic inflammatory skin disease characterized by widespread scaly erythema or plaques, often accompanied by varying degrees of itching and skin damage [1]. Globally, approximately 125 million people are affected by psoriasis, which imposes a substantial physical and psychological burden on patients, often leading to depression, anxiety, and even suicidal tendencies [2, 3, 4]. Despite continuous advances in therapeutic approaches, significant challenges remain, including suboptimal efficacy in certain patients and the occurrence of adverse effects. Therefore, there is an urgent need to develop more effective and safer treatment strategies for psoriasis.

Treatment options for psoriasis include topical drug therapies, phototherapy, systemic agents (e.g., methotrexate), and biologics targeting proinflammatory cytokines [5, 6, 7, 8]. Topical therapies remain the mainstay due to their reduced risk of systemic side effects and improved patient compliance [9]. Methotrexate (MTX), an antifolate drug, is currently regarded as a primary treatment option for various inflammatory conditions [10]. MTX acts as an immunosuppressive agent, playing a key role in modulating the inhibition of pathogenic cells and cytokines, thus suppressing immune responses [11]. However, the oral administration of MTX has significant limitations, including the first‐pass effect in the liver, which reduces its efficacy, as well as adverse side effects such as nausea and vomiting [12, 13]. Subcutaneous injection avoids these drawbacks of oral administration, but the associated pain can lead to poor patient adherence [14]. Furthermore, due to the rich vascularity and loose tissue structure of the subcutaneous space, MTX is rapidly cleared and metabolized, leading to a reduced therapeutic effect and requiring frequent dosing [15, 16]. Thus, there is a need for alternative administration strategies to enhance the therapeutic effect of MTX while minimizing its adverse reactions.

During the course of psoriasis, most patients experience itching, which significantly impacts their quality of life [17, 18]. Itching and scratching can result in Koebner phenomena, with the formation of new psoriatic plaques, further exacerbating psoriatic symptoms [19, 20]. Therefore, in the treatment of psoriasis, alleviating psoriatic itch is a crucial issue for improving the management of the condition. In addition, the excessive proliferation of keratinocytes, immune cell infiltration, and skin inflammation damage the epidermal barrier [21, 22, 23]. Dysbiosis of the skin microbiome, along with scratching, may further exacerbate skin damage, significantly increasing the risk of skin infections. Therefore, the treatment plan for psoriasis should include both antibacterial and antipruritic measures.

Zinc, an essential trace element for life, serves as a vital cofactor for numerous biological enzymes and plays a key role in a wide range of physiological processes. Moreover, in addition to its strong bactericidal effects against common bacteria, zinc also exhibits remarkable efficacy against multidrug‐resistant strains [24, 25, 26]. It is therefore often incorporated into biomaterials for the treatment of wound infections [27, 28]. The primary antibacterial mechanism of Zn^2^^+^ involves the generation of reactive oxygen species (ROS), which induce oxidative damage to bacterial DNA, enzymes, proteins, and other cellular components, thereby disrupting bacterial replication. In addition, zinc ions can adhere to bacterial membranes, penetrate them, and cause membrane rupture, leading to leakage of cytoplasmic contents and ultimately resulting in bacterial death [29, 30, 31]. Difelikefalin, a selective peripherally acting kappa opioid receptor (KOR) agonist, was first approved in 2021 for the treatment of moderate‐to‐severe pruritus in adults undergoing hemodialysis. Difelikefalin is a selective peripherally acting kappa opioid receptor (KOR) agonist [32, 33]. By activating KORs on peripheral sensory neurons, it inhibits the afferent transmission of sensory signals to the central nervous system [34]. This compound has a low potential for addiction [35]. In August 2021, Difelikefalin (DFK) was approved by the U.S. Food and Drug Administration (FDA), and in April 2022, it received approval from the European Medicines Agency (EMA), becoming the first drug approved for the treatment of skin itching in adult hemodialysis patients [36, 37].

**FIGURE 1:**
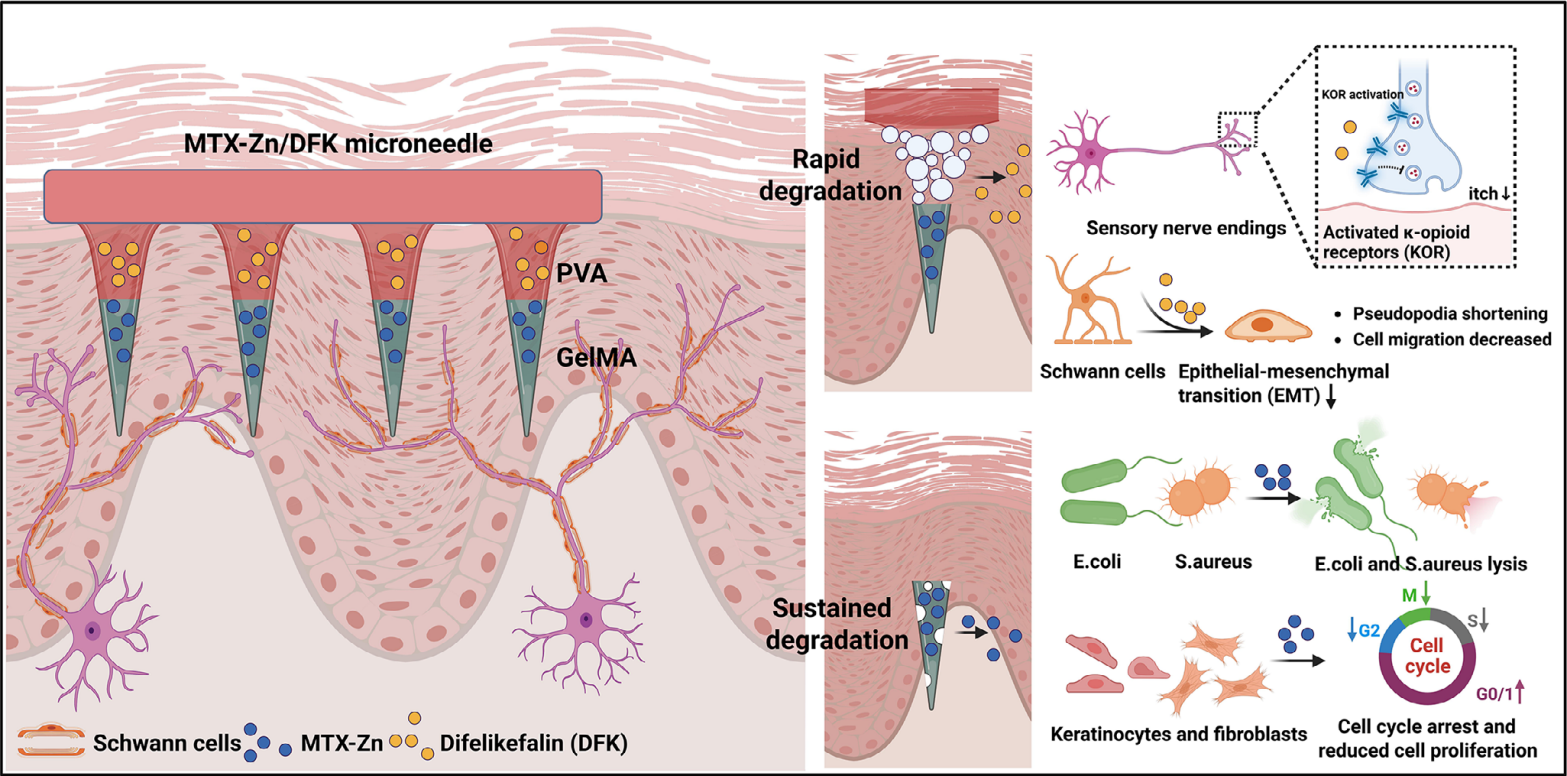
Working model illustrating the spatiotemporal drug release and therapeutic mechanisms of MTX‐Zn/Difelikefalin dual‐layer microneedle.

Transdermal drug delivery offers an attractive alternative route for treating dermatological diseases [36, 37]. This method enables direct targeting of the lesion site on the skin, thus minimizing the adverse side effects associated with systemic drug exposure and enhancing patient compliance. Microneedle (MN) technology represents a promising approach for transdermal drug delivery, offering benefits such as painlessness, reduced invasiveness, and improved efficiency [38]. MN patches have been shown to effectively deliver a range of drugs for the treatment of psoriasis, achieving superior efficacy compared to oral administration. For instance, Zhu et al. developed dexamethasone‐loaded cationic liposome dissolvable MNs, demonstrating significant therapeutic efficacy for psoriasis [39]. Donnelly et al. developed dissolving MNs loaded with MTX nanocrystals, significantly enhancing localized intradermal delivery for effective psoriasis treatment [40]. However, in studies on microneedle‐based psoriasis treatment, little attention has been paid to psoriasis‐related complications such as skin pruritus and the risk of skin infections. Therefore, in this study, we first synthesized a methotrexate‐zinc compound that combines the therapeutic effects of methotrexate for psoriasis and the antimicrobial properties of zinc ions through a coordination reaction. Polyvinyl alcohol (PVA) and Methacrylated gelatin (GelMA), both of which exhibit excellent biocompatibility and drug‐loading capacity, were used as materials for microneedle fabrication. PVA was used as the base for the microneedles, carrying difelikefalin, while GelMA was used to form the microneedle tips, containing methotrexate‐zinc. Based on the degradation characteristics of PVA and GelMA, PVA rapidly degrades, releasing difelikefalin to provide fast relief from acute itching. Meanwhile, the GelMA microneedle tips remain in the skin, and GelMA degrades slowly, releasing methotrexate‐zinc for sustained treatment of psoriasis and its antimicrobial effects (as shown in Figure 1). Our study introduces a novel microneedle‐based delivery system that combines effective treatment for psoriasis with targeted anti‐itch and antimicrobial effects, offering a promising strategy for improving patient outcomes in psoriasis management.

## 2 Results and Discussion

### 2.1 Structure, Composition, and Characteristics of MTX‐Zn

**FIGURE 2:**
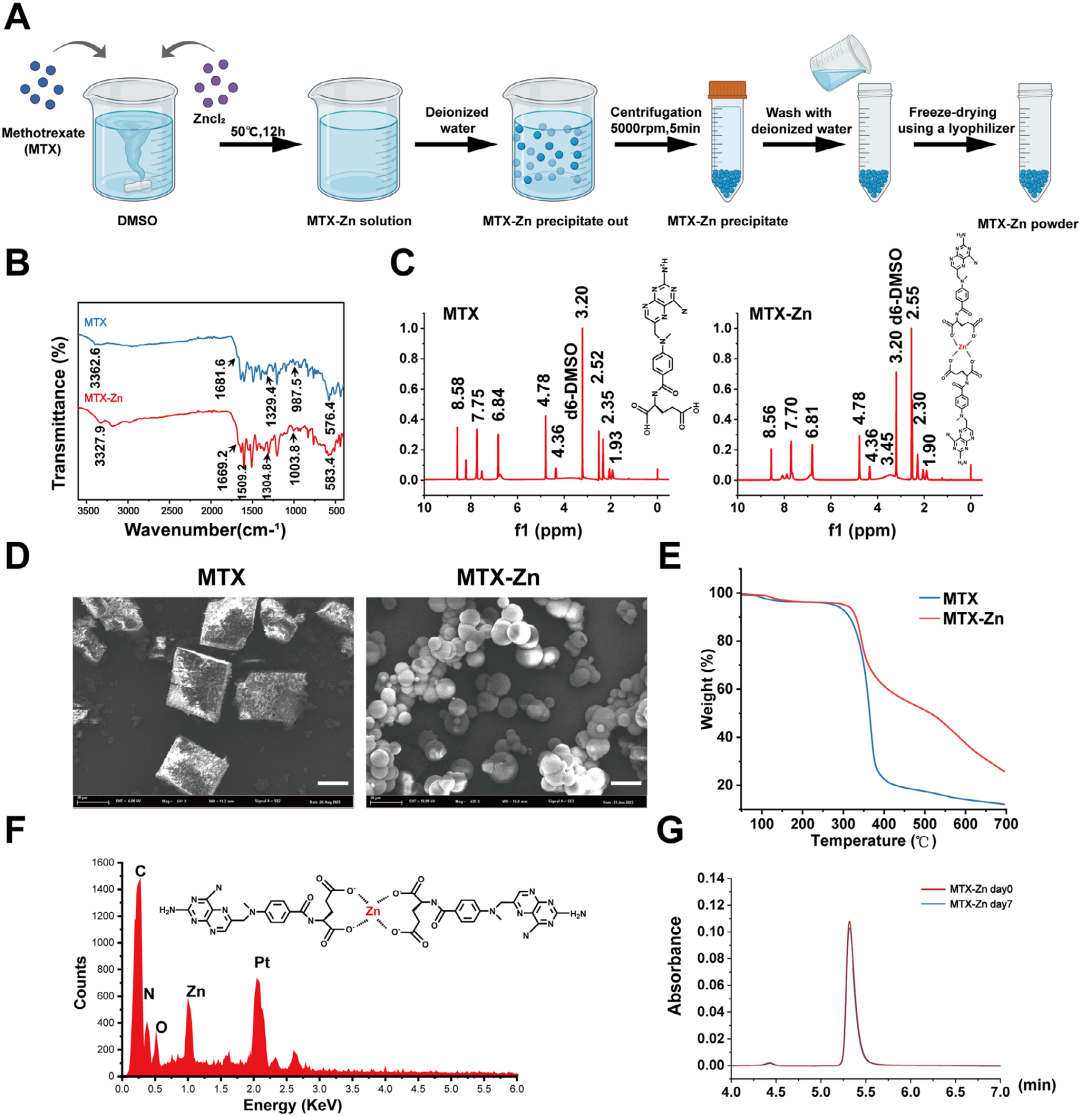
Characterization of the MTX‐Zn coordination complex. (A) Schematic illustration of the preparation process of the MTX‐Zn complex. (B) FTIR spectra of MTX and MTX‐Zn. (C) ^1^H NMR spectra of MTX and MTX‐Zn. (D) SEM images of MTX and MTX‐Zn. (E) TGA analysis of MTX and MTX‐Zn. (F) EDS spectrum. (G) HPLC analysis showing the chemical stability of MTX‐Zn over seven days, with no significant degradation detected.

Coordination compounds play a pivotal role in modern inorganic and bioinorganic chemistry and have demonstrated significant therapeutic potential in fields such as antibacterial and anticancer applications. Benefiting from the synergistic interaction between metal centers and organic ligands, metal‐organic coordination products integrate the advantages of both components and exhibit superior functional properties [41, 42]. In this study, the MTX‐Zn complex was obtained through the coordination reaction between ZnCl_2_ and methotrexate (MTX), in accordance with the fundamental principles of metal‐organic coordination chemistry (Figure 2A). The mass ratio of Zn^2^^+^ to MTX in the synthesized MTX‐Zn complex was measured to be 1:6.05 by ICP‐MS, further confirming the successful formation of the MTX‐Zn coordination complex (Table 1).

**TABLE 1:** Zn^2+^/MTX ratio in MTX‐Zn determined by ICP‐MS.

	**MTX‐Zn Weight (mg)**	**MTX‐Zn Volume (L)**	**Zn2+ Conc. (ppb)**	**Zn2+ Conc. CPS**	**Zn2+ Mass (mg)**	**Zn2+/MTX (mg/mg)**	**Zn2+/MTX (mM/mM)**
repeat1	10.0	8	178.834	124223.88	1.431	0.167	1.16076
repeat2	15.0	8	269.818	187210.82	2.159	0.168	1.16076
repeat3	20.0	8	345.814	239822.25	2.767	0.161	1.11604

The FTIR spectra of MTX and MTX‐Zn complex exhibit distinct differences, confirming the successful coordination between MTX and Zn^2+^ (Figure 2B). In the spectrum of free MTX, a broad absorption band at 3362.6 cm^−1^ corresponds to the stretching vibration of ─OH and ─NH groups. After coordination with Zn^2+^, this band shifts slightly to 3327.9 cm^−1^, indicating the involvement of these groups in metal binding. The characteristic C═O stretching vibration of MTX at 1681.6 cm^−1^ shows a noticeable shift to 1669.2 cm^−1^ in MTX‐Zn, suggesting the participation of the carboxylate group in coordination. Additionally, the aromatic and heterocyclic vibrations at 1329.4 cm^−1^ and 987.5 cm^−1^ in MTX also shift to 1304.8 cm^−1^ and 1003.8 cm^−1^, respectively, further supporting structural changes induced by Zn^2+^ binding. Importantly, a new absorption peak appears at 576.4–583.4 cm^−1^, which is typically assigned to M–O (metal–oxygen) stretching, providing direct evidence of Zn–O coordination. Together, these spectral changes demonstrate that MTX successfully complexes with Zn^2+^ to form the MTX‐Zn structure.

The ^1^H NMR spectra of MTX and the MTX‐Zn complex were compared as an additional method to verify the coordination reaction between ZnCl_2_ and methotrexate (MTX) (Figure 2C). In the spectrum of MTX, characteristic aromatic proton peaks are observed at δ 8.58 ppm, 7.75 ppm, and 6.84 ppm, while the aliphatic protons appear at δ 4.78 ppm, 4.36 ppm, 2.52 ppm, 2.35 ppm, and 1.93 ppm, consistent with the reported proton assignments of MTX. After coordination with Zn^2+^, the MTX‐Zn spectrum retains the same overall proton pattern, indicating that the fundamental molecular framework of MTX remains intact. However, multiple proton signals exhibit slight chemical‐shift changes, such as δ 8.58 → 8.56 ppm, 7.75 → 7.70 ppm, 6.84 → 6.81 ppm, and variations in the aliphatic region (e.g., 4.36 → 4.45 ppm and 2.52 → 2.55 ppm). These shifts reflect alterations in the electronic environment around the aromatic and carboxylate‐adjacent protons caused by Zn^2+^ binding. No new proton peaks were detected, but the consistent and systematic shifts across several proton environments indicate that MTX successfully coordinates with Zn^2+^, primarily through its carboxylate groups. Together, the observed changes in the ^1^H NMR spectrum confirm the successful synthesis of the MTX‐Zn complex.

Previous reports indicate that coordination of organic ligands and metal ions can lead to changes in the morphology and size of the resulting complexes. The morphology of MTX and the MTX‐Zn complex was analyzed using SEM to investigate their structural differences after coordination [43]. The SEM image of MTX shows well‐defined, square‐shaped crystalline particles (Figure 2D). The scale bar in the image indicates that the size of these crystals is relatively large, exhibiting a more rigid and structured morphology. In contrast, the SEM image of the MTX‐Zn complex reveals a significant change in morphology. The MTX‐Zn particles appear as smaller, more regularly shaped spheres. This change in particle shape and size suggests that the coordination of Zn^2+^ with MTX alters the crystal structure, likely due to the formation of a more amorphous or loosely packed structure. This morphological transformation further supports the successful synthesis of the MTX‐Zn complex.

The thermogravimetric analysis (TGA) results indicated that MTX underwent a sharp weight loss between approximately 350–400°C, corresponding to its thermal decomposition. In contrast, the MTX‐Zn complex exhibited an onset of weight loss at around 400°C, but the rate of decomposition was slower and more gradual compared to MTX (Figure 2E). The slower thermal decomposition of MTX‐Zn relative to MTX suggests that the thermal stability of MTX is significantly enhanced by the complexation with Zn ions. EDS analysis was performed to confirm the elemental composition of the MTX‐Zn complex. As shown in the spectrum, characteristic peaks corresponding to C, N, and O were observed. Notably, a clear and distinct Zn peak appeared at approximately 1.0 keV, confirming the successful incorporation of zinc ions into the MTX structure (Figure 2F). The presence of the Zn signal provides direct evidence that MTX effectively coordinates with Zn^2+^ to form the MTX‐Zn complex. HPLC analysis demonstrated that MTX‐Zn remained chemically stable over seven days. The chromatograms for day 0 and day 7 were almost completely superimposable. No additional impurity peaks or notable changes in absorbance were detected. These findings indicate that MTX‐Zn maintained its structural integrity and did not undergo detectable degradation during the seven‐day storage period (Figure 2G).

### 2.2 The Effects of MTX‐Zn on Cell Proliferation

Keratinocytes and fibroblasts are two important cell types involved in the pathogenesis of psoriasis [44]. Methotrexate (MTX) is a folate analog that inhibits cell proliferation by blocking dihydrofolate reductase (DHFR), disrupting nucleotide synthesis and DNA replication [45, 46]. Here, we first examined whether the introduction of zinc ions would affect the inhibitory effect of MTX on cell proliferation.

**FIGURE 3:**
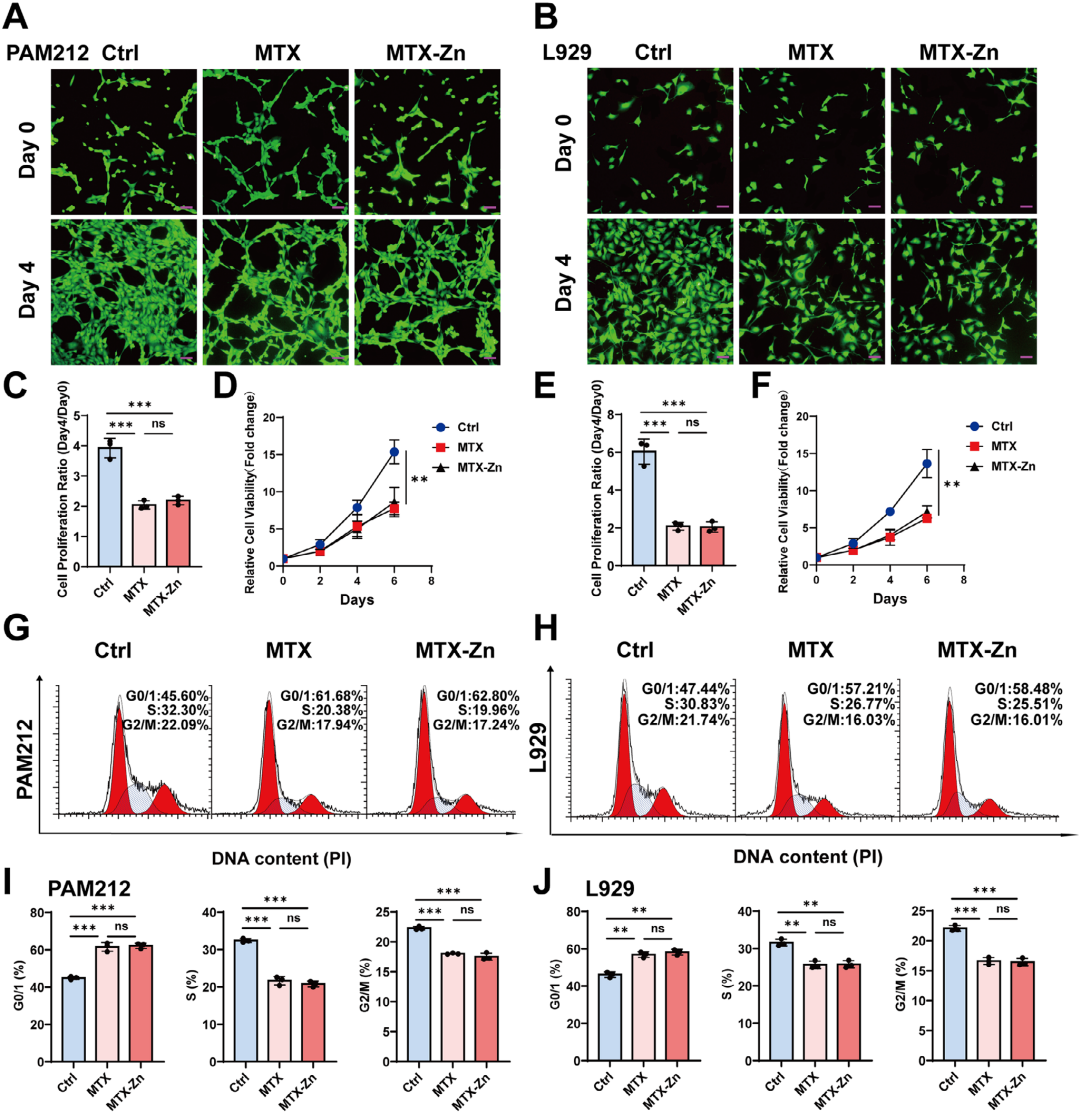
Effects of MTX and MTX‐Zn on keratinocyte and fibroblast proliferation and cell‐cycle progression. (A) Representative fluorescence images of PAM212 keratinocytes treated with Ctrl, MTX, or MTX‐Zn at Day 0 and Day 4. (B) Representative fluorescence images of L929 fibroblasts under the same treatments and time points. (C, E) Quantification of cell proliferation ratio (Day 4/Day 0) in PAM212 (C) and L929 (E) cells. (D,F) CCK‐8 assay showing relative cell viability of PAM212 (D) and L929 (F) cells over 6 days under different treatments. (G, H) Flow cytometry analysis of cell cycle distribution in PAM212 (G) and L929 (H) cells following treatment with Ctrl, MTX, or MTX‐Zn. (I,J) Statistical analysis of the percentages of G0/G1, S, and G2/M phase cells in PAM212 (I) and L929 (J) derived from flow cytometry data. Data are presented as mean ± SD. ns, not significant; ^**^ *p* < 0.01, ^***^ *p* < 0.001.

We first treated keratinocytes with different concentrations of MTX‐Zn and found that when the concentration of MTX‐Zn exceeded approximately 10 µm, the OD value in the CCK8 assay significantly decreased (Figure S1A). While MTX induces DNA damage and cell cycle arrest, it may also initiate mild apoptosis. In contrast, the toxic effects of high‐dose MTX cause more pronounced cell apoptosis. Therefore, to determine whether the slowed cell proliferation induced by MTX is due to the drug's pharmacological action or its toxicity, we performed flow cytometry experiments. In the flow cytometry apoptosis assay, a small amount of cell apoptosis was detected at a concentration of 10 µm MTX‐Zn (Figure S1B,C). However, when the concentration of MTX‐Zn exceeded approximately 10 µm, a significant increase in cell apoptosis was observed. Therefore, we conclude that the inhibition of cell proliferation induced by MTX‐Zn at concentrations above 10 µm is likely primarily due to the drug's toxicity. Based on these findings, 10 µm was selected as the concentration for subsequent in vitro experiments. Through fluorescence microscopy, we found that 10 µm MTX‐Zn exerted a pronounced inhibitory effect on the proliferation of both keratinocytes and fibroblasts, with no obvious difference compared with MTX (Figure 3A–C,E). A similar situation was also observed in the experimental results of cell proliferation by CCK8 assay (Figure 3D,F). Both MTX‐Zn and MTX induced a marked arrest in the cell cycle (Figure 3G–J). Flow cytometry cell cycle analysis showed that the proportion of cells in the G0/G1 phase was significantly increased, while the fractions of cells in the S and G2/M phases were remarkably decreased following MTX‐Zn or MTX treatment.

### 2.3 Bacteriostatic Effect of MTX‐Zn

**FIGURE 4:**
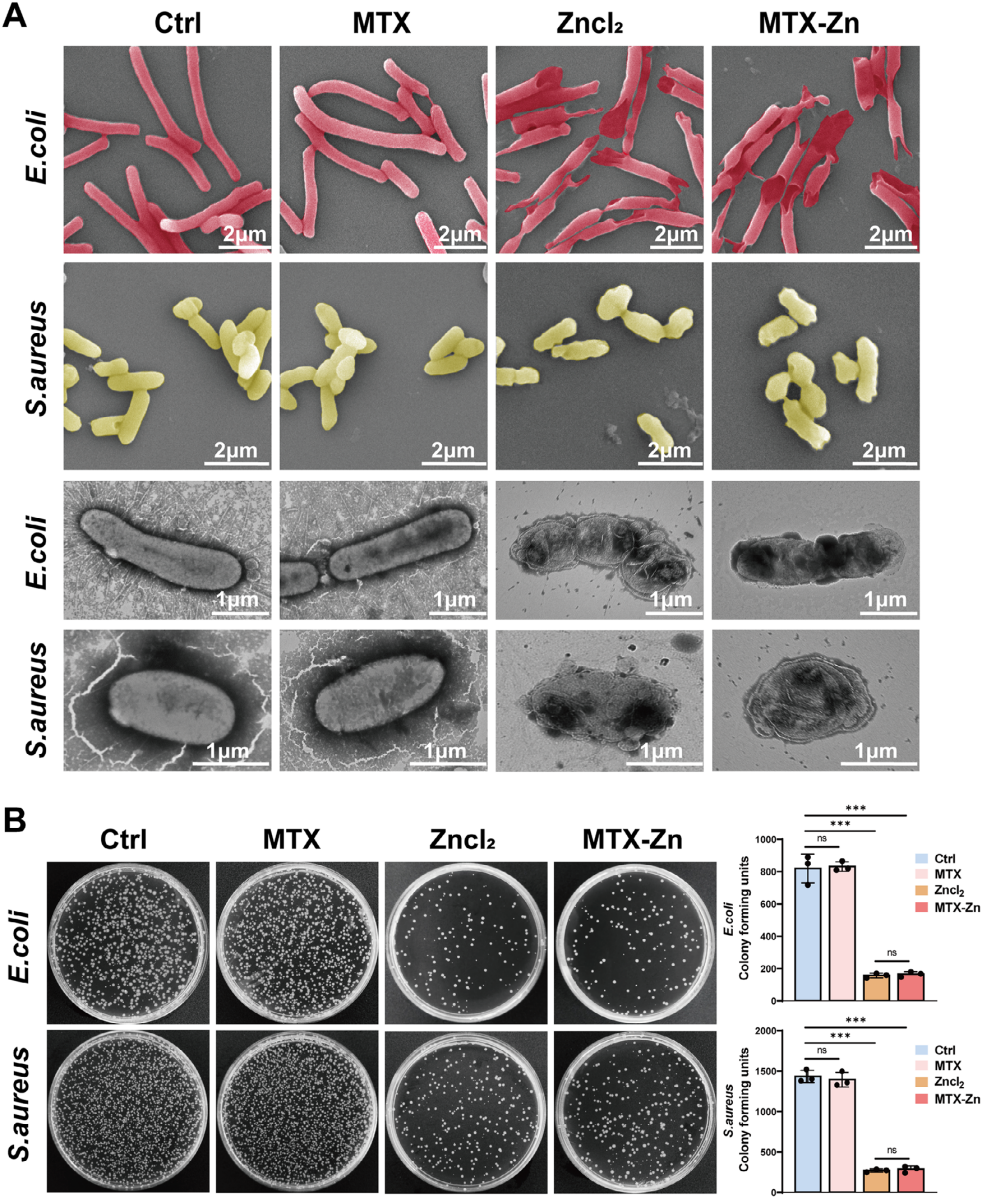
Effect of MTX‐Zn on bacterial morphology and colony formation. (A) Scanning electron microscopy (SEM) and transmission electron microscopy (TEM) images showing the morphological changes in *E. coli* and *S. aureus* under different treatments. (B) Colony formation assay demonstrating the effect of MTX‐Zn on bacterial growth. The graph on the right quantifies the number of colony‐forming units (CFUs) for *E. coli* and *S. aureus*. Data are presented as the mean ± SD. ns, not significant; ^**^ *p* < 0.01, ^***^ *p* < 0.001.

Zinc ions (Zn^2^^+^) exert inhibitory effects on bacteria through multiple mechanisms. First, Zn^2^^+^ ions can disrupt the integrity of the bacterial cell membrane, leading to the leakage of cytoplasm and causing bacterial shrinkage or death. Secondly, zinc ions can induce the generation of reactive oxygen species (ROS), which further exacerbate bacterial death. Additionally, Zn^2^^+^ interferes with the bacterial enzyme system, inhibiting its metabolic activity and, consequently, suppressing bacterial growth and proliferation [30, 47]. To evaluate whether the MTX‐Zn complex retains the antibacterial properties of zinc ions, we assessed its bacteriostatic effect against typical Gram‐negative and Gram‐positive bacteria, *Escherichia coli* (*E. coli*) and *Staphylococcus aureus* (*S. aureus*), following the method described in previous literature [48]. As shown in Figure 4A, both *E. coli* and *S. aureus* treated with MTX and MTX‐Zn exhibited significant morphological changes, including surface irregularities, cell membrane rupture, and collapse. In contrast, the control group displayed normal rod‐shaped structures for E.coli and typical coccoid forms for S.aureus. To quantitatively analyze the antimicrobial activity of MTX‐Zn, we performed a bacterial plate cloning experiment. As shown in Figure 4B, the bacteriostatic effect of 10 µm MTX‐Zn was similar to that of ZnCl_2_ on both E.coli and S.aureus. The number of colonies was significantly reduced in both the ZnCl_2_ and MTX‐Zn groups compared to the control group. Collectively, for both bacteria, MTX‐Zn exhibited the best antibacterial properties, indicating that the MTX‐Zn complex, formed by the coordination of Zn^2^^+^ with MTX, retains the antimicrobial activity of zinc ions.

### 2.4 Preparation and Characterization of MZ/D‐DL‐MN

To achieve long‐lasting therapeutic effects for psoriasis and the rapid anti‐itch effect of microneedles, we chose Gelatin methacryloyl (GelMA) and polyvinyl alcohol (PVA) as the materials for microneedle fabrication.

GelMA is obtained by modifying gelatin through methacryloylation, retaining the biological activity sites and enzymatically degradable sequences of gelatin. It contains abundant hydrophilic groups, giving it good water solubility [49, 50]. After UV crosslinking, GelMA shows that the smaller the pores, the denser the structure, and the slower the degradation rate [51, 52, 53]. PVA is a water‐soluble polymer material with excellent biocompatibility and degradability. Due to its biological safety, mechanical properties, and rapid dissolution, PVA is chosen as a material for making microneedles [54, 55].

**FIGURE 5:**
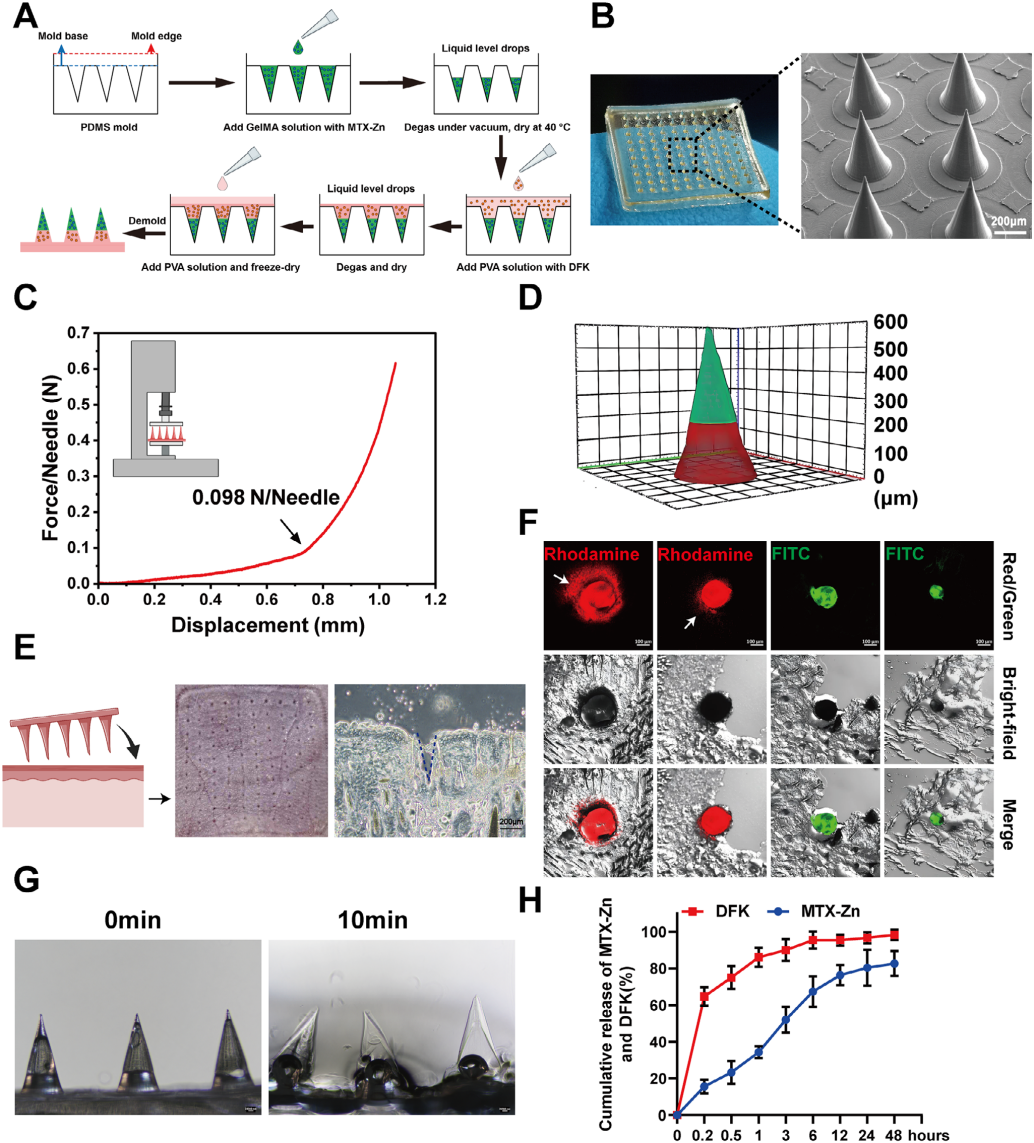
Synthesis, characterization, and release behavior of MTX‐Zn/Difelikefalin dual‐layer microneedles (MZ/D‐DL‐MN). (A) Schematic illustrating the fabrication process of the MZ/D‐DL‐MN. (B) SEM image showing the structure of the MZ/D‐DL‐MN. (C) Force‐displacement curve for mechanical strength testing of the microneedles. (D) The 3D fluorescence images show that the two drugs are separately loaded at different spatial positions within the microneedle tips (Rhodamine, red; FITC, green). (E) Skin insertion ability of MZ/D‐DL‐MNs in mice, with histological analysis of skin tissue showing successful microneedle penetration. (F) Merged fluorescence and bright‐field images further confirm the dual‐layer structure and compartmentalized drug distribution in the microneedles. The arrows indicate that the PVA portion of the microneedle tips degrades after insertion into the skin, thereby releasing the rhodamine dye encapsulated in the PVA matrix. (G) Bright‐field images showing the degradation behavior of the dual‐layer microneedles in physiological saline. The lower half of the microneedle tips undergoes noticeable dissolution, while the upper tip structure remains intact. (H) Sustained release profile of the MZ/D‐DL‐MN system.

In this study, as shown in Figure 5A, we incorporated MTX‐Zn into the GelMA solution to form the first half of the microneedle tip, while Difelikefalin, which has anti‐itch effects, was added to the PVA solution to form the second half of the microneedle tip. Upon implantation, the PVA rapidly dissolves in the body, releasing the Difelikefalin for quick anti‐itch action, while the MTX‐Zn/GelMA at the microneedle tip remains in the skin, slowly releasing MTX‐Zn to treat psoriasis.

The prepared microneedles were characterized using SEM. As shown in the SEM images, the microneedles are arranged in an orderly pattern, with a conical shape and sharp tips, ensuring sufficient skin penetration (Figure 5B). Each microneedle exhibited a failure force of 0.098N/per needle (Figure 5C). Given that the minimum force required for a microneedle to penetrate the stratum corneum is 0.058 N per needle, our microneedles possess sufficient mechanical strength to effectively penetrate the stratum corneum layer.

To further verify whether the microneedles possessed sufficient mechanical strength to penetrate the skin, in vivo skin insertion test was conducted. As illustrated in the schematic, the microneedle array was pressed onto the skin surface. The resulting tissue imprint displayed a uniformly distributed pattern of microchannels that precisely matched the arrangement of the microneedle array. Histological examination of the longitudinal tissue section further confirmed the formation of well‐defined microchannels created by the microneedles. These channels extended through the stratum corneum into the viable epidermis and dermis, demonstrating that the microneedles effectively breached the skin barrier without causing excessive tissue damage (Figure 5E).

To verify the dual‐drug loading capability of the microneedle tips, the upper region of each microneedle was loaded with fluorescein isothiocyanate (FITC), while the lower portion was loaded with rhodamine. Confocal laser scanning microscopy (CLSM) 3D imaging clearly demonstrated the distinct spatial distribution of the two fluorophores within the microneedle tip. FITC fluorescence was confined to the upper conical region, whereas rhodamine fluorescence was localized in the lower segment, confirming that the microneedle structure can successfully carry and compartmentalize two different agents within a single needle tip (Figure 5D). To assess needle integrity after insertion, microneedles were applied to the skin and examined by CLSM through sequential horizontal cryosectioned sections. The circular fluorescence patterns corresponding to the needle tips remained uniform across all layers, indicating that the microneedles penetrated the skin while maintaining their original shape without bending or deformation (Figure 5F). From this result, we can also observe that after the microneedles are inserted into the skin, the rhodamine located in the PVA layer diffuses out of the microneedles, indicating that the PVA layer of the microneedles dissolves within a short period (Figure 5F). We next evaluated the degradation behavior and drug release profile of the MZ/D‐DL‐MN. After the addition of physiological saline to the microneedles, rapid deformation and degradation of the PVA layer were observed within 10 min (Figure 5G). The release kinetics of difelikefalin (DFK) and MTX‐Zn from MZ/D‐DL‐MN system were further examined (Figure 5H). DFK exhibited a rapid initial release, with more than 80% of the drug released within the first hour, consistent with the fast dissolution of the PVA layer. In contrast, MTX‐Zn showed a gradual and sustained release pattern, with cumulative release increasing steadily over 48 h. These results confirm that the dual‐layer microneedle design enables spatiotemporally controlled dual‐drug delivery, achieving fast release of DFK for immediate antipruritic effects and sustained release of MTX‐Zn for prolonged therapeutic action.

### 2.5 In Vivo Therapeutic Efficacy of MTX‐Zn/Difelikefalin Dual‐Layer Microneedle (MZ/D‐DL‐MN) for Psoriasis Treatment

**FIGURE 6:**
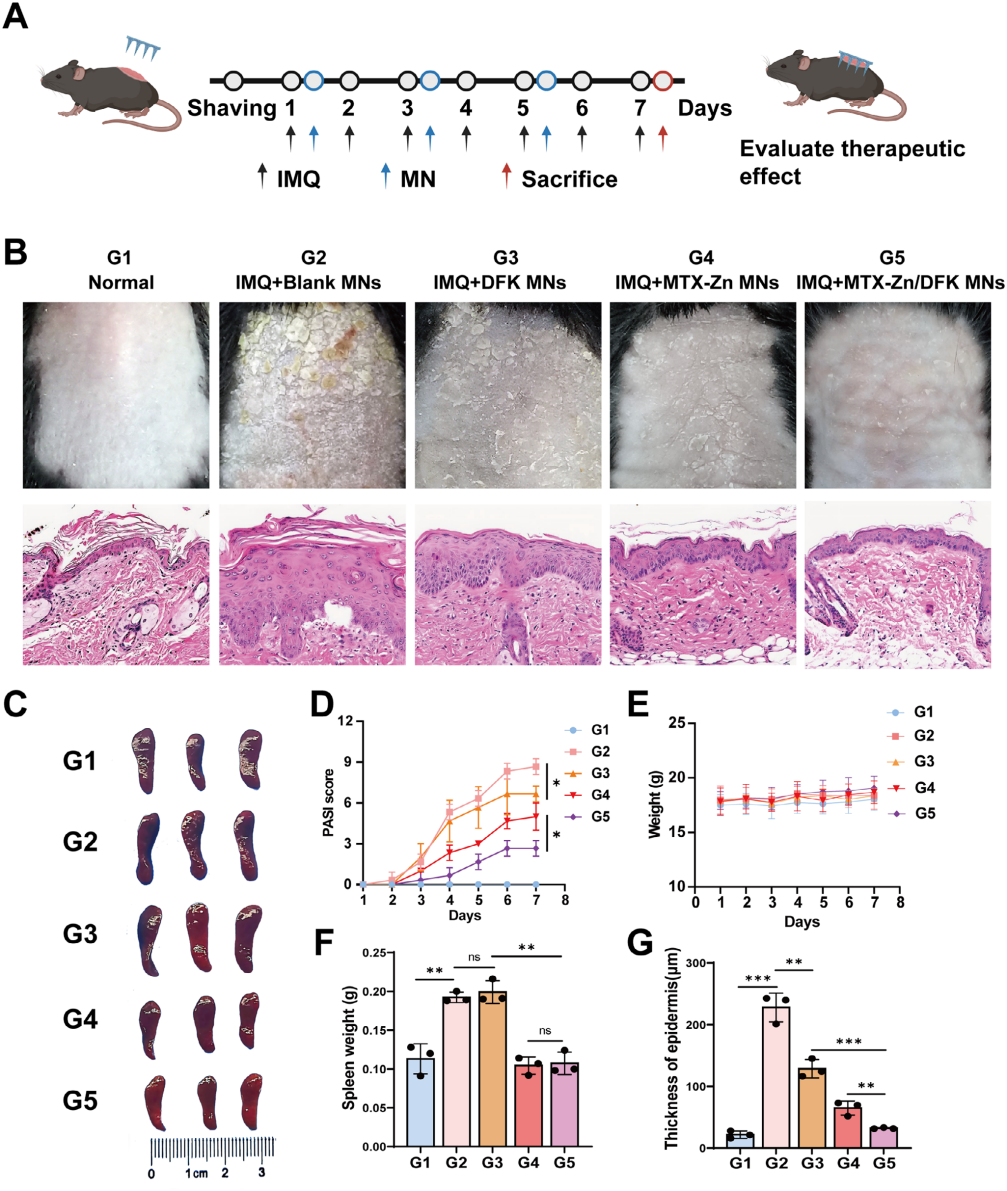
Therapeutic effects of MTX‐Zn/DFK microneedles on IMQ‐induced psoriasis‐like dermatitis in mice. (A) Schematic illustration of the experimental timeline. (B) Representative clinical photographs and HE‐stained skin sections from each group. (C) Representative images of spleens collected from each group. (D) PASI scores were recorded over the treatment period. (E) Changes in mouse body weight during treatment showed no significant systemic toxicity in any group. (F) Quantification of spleen weight. (G) Quantification of epidermal thickness. Data are presented as mean ± SD. ns, not significant; ^*^ *p* < 0.05, ^**^ *p* < 0.01, and ^***^ *p* < 0.001.

To evaluate the therapeutic effect of MZ/D‐DL‐MN on psoriasis in mice, the animal experimental procedure is outlined in Figure 6A. The therapeutic efficacy of MZ/D‐DL‐MN was evaluated using an imiquimod (IMQ)‐induced psoriasis mouse model. Skin lesions in IMQ‐induced psoriasis typically exhibit erythema, induration, and thickening [56]. By day 7, pronounced psoriasis‐like lesions characterized by severe hyperkeratosis were observed in the blank MN‐treated group (Figure 6B). Compared with this group, the difelikefalin MN‐treated, MTX MN‐treated, and MTX‐Zn MN‐treated groups showed varying degrees of improvement in clinical symptoms (Figure 6B). Among them, the MTX‐Zn/Difelikefalin MN‐treated group (MZ/D‐DL‐MN) displayed the most significant therapeutic benefit. Psoriasis Area and Severity Index (PASI) scores further confirmed that MZ/D‐DL‐MN was more effective than all other administration methods (Figure 6D). IMQ is known to induce splenomegaly [57]. After treatment, spleen weight decreased, with MTX‐loaded MNs producing a notably strong anti‐splenomegaly effect and resulting in lighter spleens compared with other groups (Figure 6C,F).

To further evaluate skin recovery, mice were euthanized, and epidermal thickness on the dorsal skin was quantified. All treatment groups exhibited different degrees of improvement in epidermal hyperplasia, with MZ/D‐DL‐MN achieving the most pronounced therapeutic effect (Figure 6G). Interestingly, although the difelikefalin MN‐treated group slightly alleviated psoriasis lesions, it showed no significant reduction in splenomegaly. In addition, no significant changes in body weight were observed during MN treatment (Figure 6E). Overall, these findings suggest that the dual delivery of MTX‐Zn and difelikefalin via the proposed Dual‐Layer Microneedle system results in a markedly enhanced therapeutic efficacy against psoriasis. Notably, no evident histopathological damage was observed in major organs, including the heart, liver, kidneys, and lungs, indicating favorable in vivo biosafety (Figure S1D). This further highlights the potential of this microneedle platform as a safe and effective strategy for clinical translation.

### 2.6 Immunological Mechanism

**FIGURE 7:**
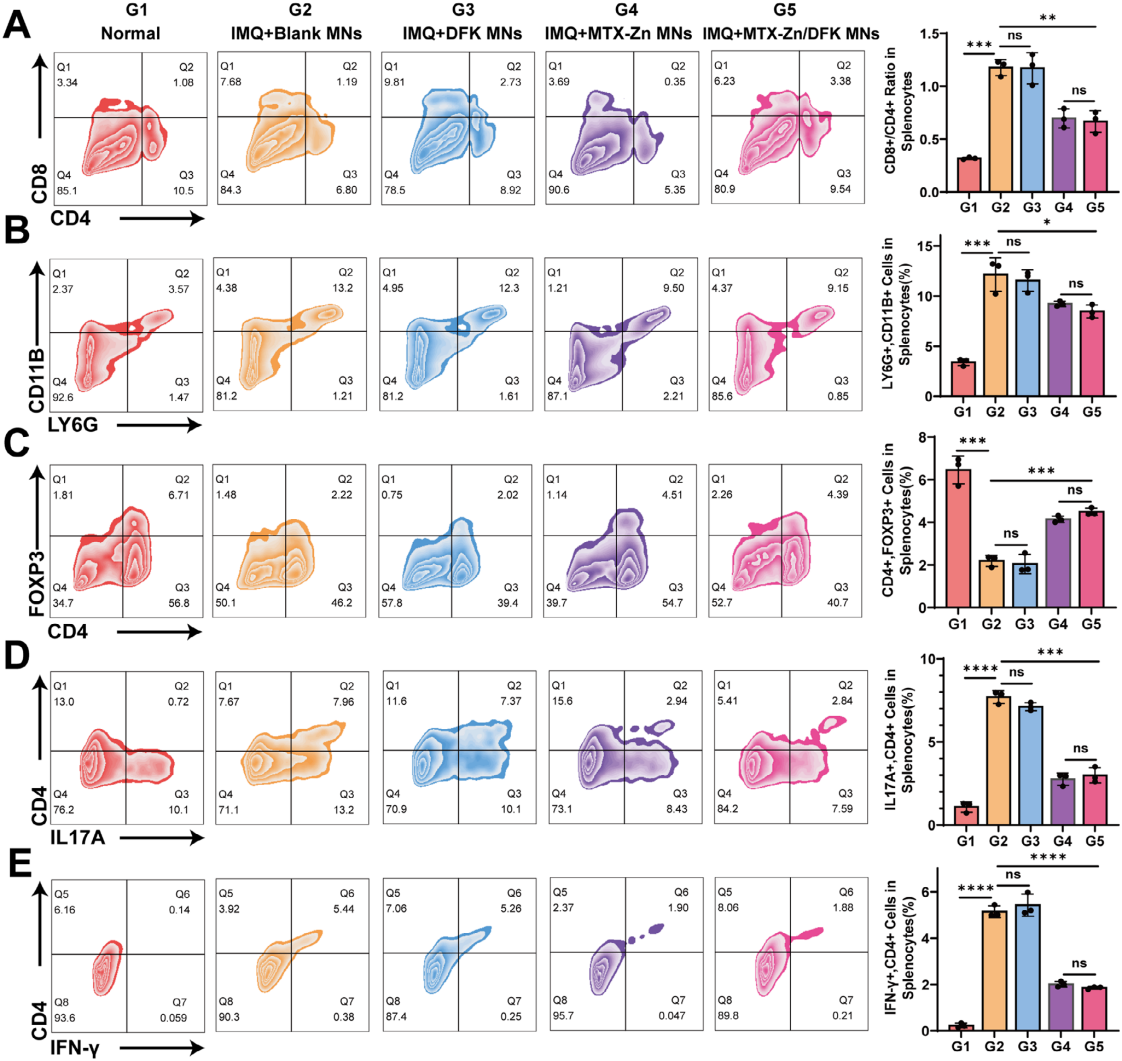
Evaluation of the various immune cells in the spleen. Representative flow cytometry analysis of T cells (CD4+, CD8+) (A), Neutrophils (LY6G+, CD11B+) (B), Tregs (CD4+, FOXP3+) (C), Th17 cells (CD4+, IL17A+) (D), and Th1 cells (CD4+, IFN‐γ+) (E), along with corresponding quantification of immune cell subsets. Data are presented as mean ± SD. ns, not significant; ^*^ *p* < 0.05, ^**^ *p* < 0.01, and ^***^ *p* < 0.001.

Psoriasis, a multisystem inflammatory disease, has long attracted substantial attention from the medical community. In recent years, considerable progress has been made in elucidating its pathogenesis and developing new therapeutic strategies [58, 59]. Previous studies have reported that IMQ administration can induce splenomegaly, which is associated with the release of inflammatory cytokines [60, 61]. To obtain a more comprehensive understanding of the alterations in splenic immune‐cell populations, we further performed flow cytometry analysis. The results show that in the IMQ‐induced psoriasis model, immune cells known to be involved in the pathogenesis of psoriasis, such as the elevated CD8^+^/CD4^+^ T cell ratio and the significantly increased neutrophil proportion (Figure 7A,B), confirm that IMQ provokes a pronounced immune‐cell imbalance within the spleen. The CD8^+^/CD4^+^ T cell ratio is an important indicator of immune homeostasis in psoriasis. An elevated ratio is generally associated with enhanced cytotoxic and inflammatory responses, contributing to disease progression [62]. In the MTX‐Zn microneedle treatment group, this immune‐cell imbalance can be effectively improved. Interestingly, the proportion of regulatory T cells (Tregs) in the spleens of mice in the MTX‐Zn microneedle treatment group was increased (Figure 7C). During the progression of psoriasis, Th17 and Th1 cells also play important roles. Th17 (CD4^+^, IL‐17A^+^) cells secrete IL‐17A/F and IL‐22, recruiting inflammatory cells and amplifying inflammation. Th1 (CD4^+^, IFN‐γ^+^) cells activate dendritic cells and macrophages by producing pro‐inflammatory cytokines such as IFN‐γ and TNF‐α, thereby enhancing antigen presentation and the inflammatory cascade.

From our results, we observed that IL‐17A^+^, CD4^+^ T cells (Th17 cells) were significantly reduced in the MTX‐Zn microneedle treatment group, showing the potential of MTX‐Zn treatment in attenuating Th17‐mediated inflammation in psoriasis (Figure 7D). Similarly, IFN‐γ^+^, CD4^+^ T cells (Th1 cells) were significantly reduced in the MTX‐Zn microneedle treatment group, highlighting the anti‐inflammatory effects of MTX‐Zn treatment (Figure 7E). These observations suggest that modulating splenic immune‐cell homeostasis may serve as a promising therapeutic approach to mitigate psoriasis progression.

In summary, the analysis of splenic immune‐cell composition revealed a substantial immune imbalance in the IMQ‐induced psoriasis model. MTX‐Zn microneedle effectively restored immune‐cell homeostasis within the spleen, offering new insights and therapeutic possibilities for psoriasis management. This study not only deepens our understanding of psoriasis pathogenesis but also provides valuable support for the development of more effective treatment strategies in the future.

### 2.7 Difelikefalin Microneedles Alleviate Pruritus in Psoriasis, Reduce the Density of Skin Nerve Terminals in Psoriasis, and Inhibit Schwann Cell Migration

**FIGURE 8:**
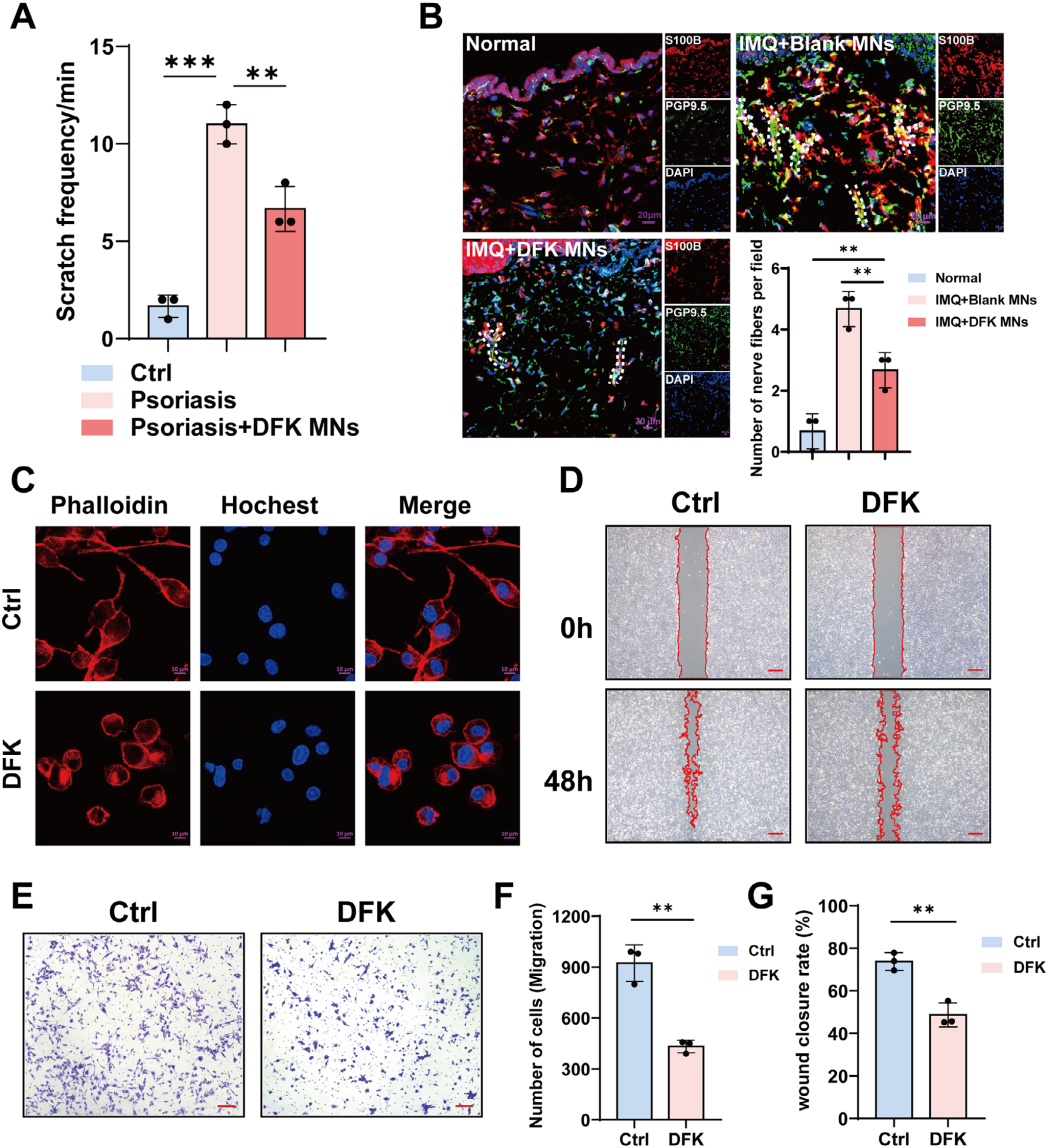
Difelikefalin microneedles alleviate pruritus in psoriasis, reduce the density of skin nerve terminals in psoriasis, and inhibit Schwann cell migration. (A) Scratch frequency measured in psoriasis‐induced mice with or without DFK MN treatment. (B) Immunofluorescence images of skin sections showing Schwann cells (S100B, green) and nerve fiber terminals (PGP9.5, red) in each group. (C) Phalloidin and Hoechst staining of the cytoskeleton and cell nuclei in Schwann cells. (D) Scratch assay to evaluate wound healing. (E) Transwell migration assay to assess Schwann cells' migration. (F) Quantification of the number of Schwann cells migrated in the transwell migration assay. (G) Quantitative analysis of wound closure rate. Data are presented as mean ± SD. ns, not significant; ^**^ *p* < 0.01, and ^***^ *p* < 0.001.

Difelikefalin (DFK) is a selective kappa‐opioid receptor agonist that alleviates pruritus by acting on peripheral sensory nerve fibers. We induced psoriasis‐like skin lesions on the mouse ears, and after successful induction, applied difelikefalin microneedles to the psoriasis‐affected areas of the mouse ears (Figure S1F). The scratching behavior of the mice on their ears was recorded in real‐time to evaluate the antipruritic effect of difelikefalin microneedles. The results showed that after IMQ‐induced psoriasis on the mouse ears, the number of ear scratches significantly increased, while treatment with difelikefalin microneedles notably reduced the scratching frequency (Figure 8A). Several studies have confirmed that in psoriasis lesions, the overactivation of nerve cells and the increase in nerve fiber density are closely associated with the exacerbation of pruritus symptoms [63, 64, 65]. As mentioned above, difelikefalin is a selective kappa‐opioid receptor agonist that alleviates pruritus by acting on peripheral sensory nerve fibers. Given the effect of difelikefalin microneedles in reducing psoriasis skin lesions, it is worth further exploring whether, in addition to activating the kappa‐opioid receptors on nerve fibers to alleviate pruritus, difelikefalin also has the potential to inhibit nerve fiber extension, thereby reducing nerve fiber density.

The immunofluorescence staining of psoriasis lesions on the mouse dorsal skin showed significant differences in the distribution of epidermal nerve fibers among the groups. In the blank MN‐treated group, psoriatic lesions showed a marked increase in dermal nerve fibers, many of which extended abnormally into the epidermis, forming dense and disordered terminal networks characteristic of neurogenic inflammation in psoriasis (Figure 8B). In contrast, treatment with DFK‐loaded microneedles significantly reduced these pathological nerve projections. The number of dermal nerve fiber terminals extending into the epidermis was noticeably decreased, and the overall nerve distribution appeared more sparse. Quantitative analysis confirmed these findings: compared with the blank MN‐treated group, the Difelikefalin MN‐treated group exhibited a significant reduction in nerve fiber counts per field (*p* < 0.01), approaching levels observed in normal skin. These results indicate that DFK‐loaded microneedle treatment effectively suppresses the abnormal epidermal extension of dermal nerve fibers associated with psoriatic inflammation.

It is well known that Schwann cells play a key role in the process of axonal extension. They promote axonal growth by migrating, secreting neurotrophic factors, and constructing “regeneration conduits” [66, 67]. We treated normally cultured Schwann cells with difelikefalin (DFK). Interestingly, through phalloidin staining, we observed a significant reduction in the pseudopodia of Schwann cells (Figure 8C). The results show that in the DFK‐treated group, Schwann cells exhibited fewer and shorter pseudopodia compared to the control group, indicating that DFK treatment reduced the extension of these cellular protrusions. The wound healing assay and transwell migration assay further supported this observation, showing a marked decrease in Schwann cell migration after DFK treatment (Figure 8D,E). The wound closure rate was significantly lower in the DFK‐treated group (*p* < 0.01) compared to the control, and the number of migrating cells in the transwell assay was also significantly reduced (*p* < 0.01) (Figure 8F,G). These results suggest that DFK treatment inhibits Schwann cell pseudopodia extension and cellular migration.

### 2.8 RNA Transcriptome Sequencing Revealed That Difelikefalin Inhibits Schwann Cell Epithelial‐Mesenchymal Transition (EMT)

**FIGURE 9:**
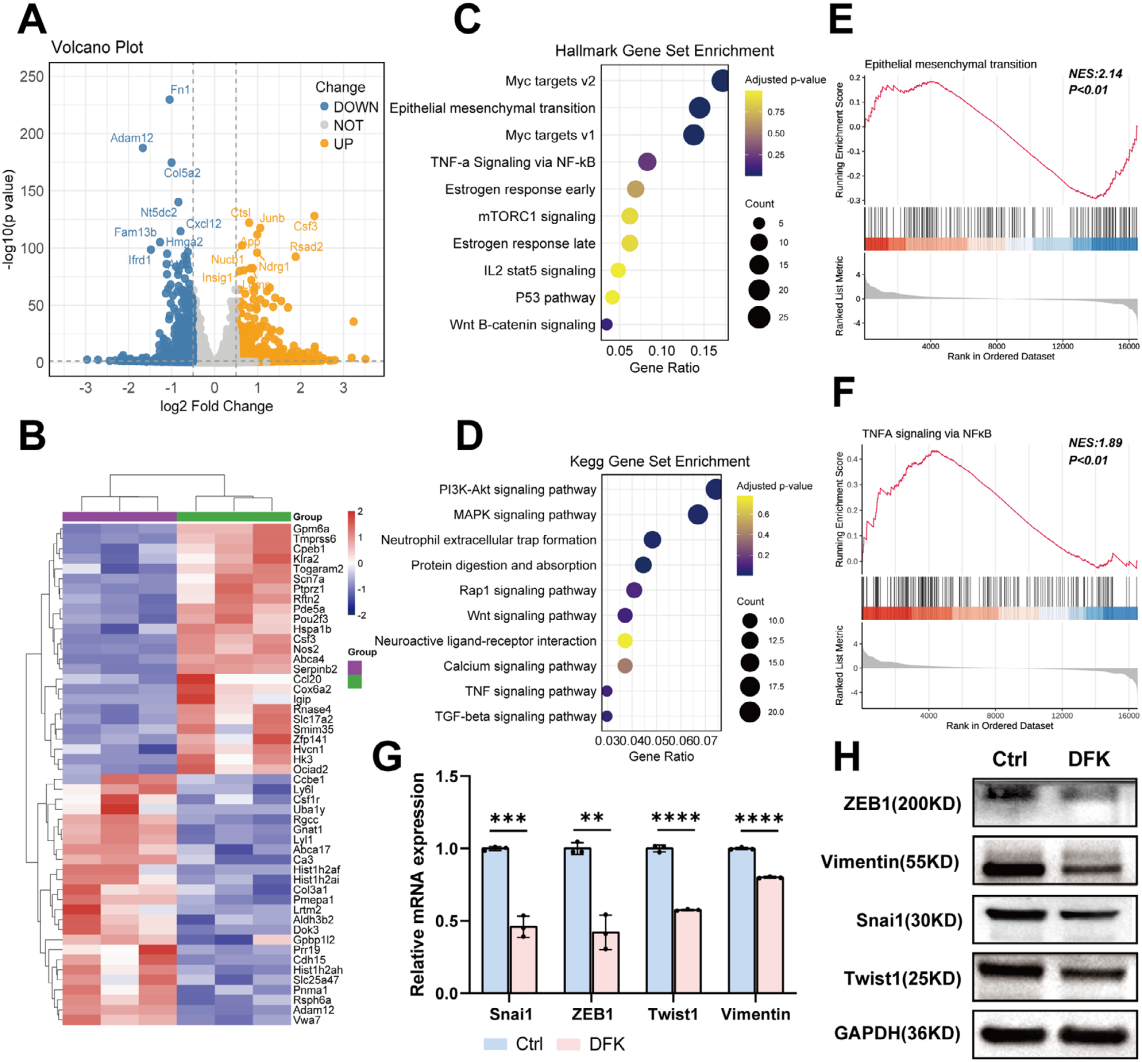
RNA transcriptome sequencing of Schwann cells treated with difelikefalin. (A) Volcano plot displaying differentially expressed genes (DEGs) between control (Ctrl) and DFK‐treated groups. (B) Heatmap of differentially expressed genes (DEGs) showing distinct transcriptional profiles between Ctrl and DFK‐treated groups. The heatmap displays the top 25 upregulated and top 25 downregulated genes ranked by fold change. (C) Hallmark gene set enrichment analysis of downregulated genes in Schwann cells treated with DFK, revealing the 9major suppressed biological pathways following treatment. (D) KEGG pathway enrichment analysis of downregulated genes in Schwann cells treated with DFK, revealing the major suppressed biological pathways following treatment. (E, F) Representative GSEA enrichment plots of downregulated genes. (G) Quantitative real‐time PCR (qPCR) analysis showing relative mRNA expression of EMT‐related genes in Ctrl and DFK groups. (H) Western blot analysis of EMT markers in Ctrl and DFK groups. Data presented as mean ± SD. ^**^ *p* < 0.01, ^***^ *p* < 0.001, ^****^ *p* < 0.0001.

We further performed transcriptome sequencing on Schwann cells treated with Difelikefalin. We identified 832 downregulated genes and 967 upregulated genes (Figure 9A,B). Upon conducting Hallmark Gene Set Enrichment Analysis and KEGG Gene Set Enrichment Analysis on the downregulated genes, we found that the Epithelial‐Mesenchymal Transition (EMT) pathway was among the top two enriched signaling pathways (Figure 9C–F). Other signaling pathways enriched with downregulated genes included TNF signaling via NF‐κB, Wnt signaling, and the PI3K‐Akt signaling pathway, all of which are closely involved in the regulation of EMT. TNF/NF‐κB signaling is known to promote EMT by inducing pro‐inflammatory transcriptional programs and enhancing mesenchymal marker expression. Wnt signaling plays a critical role in driving EMT through activation of β‐catenin‐dependent transcription, thereby facilitating cellular dedifferentiation and migration. Similarly, the PI3K‐Akt pathway contributes to EMT by regulating cell survival, cytoskeletal remodeling, and motility. These findings suggest that difelikefalin treatment reduced the EMT capacity of Schwann cells. Furthermore, we validated the results through qPCR and Western blotting, which confirmed that the mRNA and protein expression levels of classic EMT markers were decreased in difelikefalin‐treated schwann cells (Figure 9G,H).

## 3 Conclusions

In this study, we developed a dual‐layer microneedle system that combines rapid antipruritic therapy with sustained anti‐hyperproliferative, immunomodulatory, and antibacterial effects for psoriasis management. The fast‐dissolving PVA layer enabled immediate release of difelikefalin, rapidly relieving itch and reducing abnormal epidermal nerve‐fiber density, partly by inhibiting Schwann‐cell migration and EMT. The GelMA microneedle tips provided prolonged intradermal delivery of the MTX‐Zn coordination complex, which preserved the immunomodulatory properties of MTX and the antimicrobial activity of Zn^2^^+^. In an IMQ‐induced psoriasis model, this dual‐drug microneedle system significantly improved disease severity, reduced epidermal hyperplasia and splenomegaly, and restored immune‐cell homeostasis. Overall, this minimally invasive platform offers a comprehensive therapeutic strategy that simultaneously targets pruritus, epidermal hyperproliferation, immune dysregulation, and infection risk in psoriasis. Notably, this study is currently limited to preclinical evaluation in animal models. Further investigations, including long‐term safety assessment, validation in large‐animal models, and clinical studies, will be required to support future clinical translation.

## 4 Experimental Section

### 4.1 Materials

Methotrexate (MW = 454.44 Da) and zinc chloride were obtained from Macklin Company. Gelatin methacryloyl (GelMA) and polyvinyl alcohol (PVA) were obtained from Suzhou Yongqinquan Intelligent Equipment Co., Ltd. All materials and reagents were not further purified unless otherwise stated. Deionized (DI) water (>18.2 MΩ cm) was used in this study. Difelikefalin was obtained from TargetMol Co., Ltd., Boston, USA. The essential reagents for cell culture included 1% penicillin streptomycin solution (PS), fetal bovine serum (FBS), trypsin‐EDTA solution, and Dulbecco's Modified Eagle Medium (DMEM), all of which were obtained from Gibco, New York, USA. The cell lines employed in this study included mouse fibroblast cell lines (L929), Schwann cell lines (RSC96), and mouse keratinocytes (PAM212), all of which were obtained from Servicebio Biotechnology Co., Ltd., Wuhan, China. Furthermore, the following key reagent kits were procured for the execution of molecular biology experiments: SYBRGreen PreMix Pro Taq HS for quantitative polymerase chain reaction (qPCR), Evo M‐MLV RT qPCR Premix Kit, and AG RNAex Pro RNA Kit for efficient RNA extraction. These kits were obtained from Accurate Biology, Hunan, China.

### 4.2 Synthesis and Characterization of MTX‐Zn Complex

Methotrexate (MTX) and zinc chloride (ZnCl_2_) were separately dissolved in DMSO. The two solutions were then mixed to obtain a feeding molar ratio of Zn^2^^+^:MTX greater than 2:1, ensuring that an excess of ZnCl_2_ was present to drive complete coordination of MTX. The reaction mixture was maintained at 50°C under continuous magnetic stirring for 12 h. Deionized water was then gradually added to the reaction system until microcrystals precipitated. The MTX‐Zn coordination product was collected by centrifugation at 5000 rpm for 5 min, washed twice with deionized water to remove unreacted ZnCl_2_, and subsequently lyophilized to yield the MTX‐Zn powder. The attenuated total reflection (ATR) patterns of methotrexate (MTX) and MTX‐Zn were characterized by Fourier Transform Infrared Spectroscopy (FTIR) (PerkinElmer, Frontier) with a wavenumber from 500 to 4000cm^−1^ at room temperature. The thermal properties of the samples were evaluated by a thermogravimetric analyzer (TGA) (Gas Analyzer, TGA55). TGA data were recorded for each analyzed sample in the temperature range of 50–700°C. ^1^H NMR (nuclear magnetic resonance) analyses of MTX and MTX‐Zn were performed using nuclear magnetic resonance spectroscopy (Bruker, AVANCE III 500 MHz) and a n2‐cryoo‐platform for the Prodigy probe. Samples were dissolved in d6‐DMSO solvent. The experimental procedure was carried out at room temperature. Data acquisition was performed with Bruker's TopSpin 3.2 software and processed with MestReNova 14.1 (Mestrelab Research S.L.). The stability of MTX‐Zn was tested by high‐performance liquid chromatography (HPLC) (Agilent 1260 Infinity) with a diode array detector for the content and purity of the MTX‐Zn solution at day 0 and 7. Using a scanning electron microscope (SEM) (ZEISS Merlin) to observe the morphology of the MTX and MTX‐Zn at an accelerating voltage of 10 kV, and analyzing the surface elemental composition of the MTX‐Zn by energy dispersive spectrometer (EDS) (EDAX Octane Pro). All samples were sputter‐coated with platinum before observation. The zinc content in MTX‐Zn was determined using inductively coupled plasma mass spectrometry (ICP‐MS) (Agilent 7700X).

### 4.3 Cell Proliferation

Dulbecco's Modified Eagle Medium (DMEM) supplemented with 10% fetal bovine serum, 50U/mL penicillin G, and 50 µg/mL streptomycin sulfate was used for the culture of mouse fibroblasts and keratinocytes. Cells were seeded into 96‐well plates at a density of 1 × 10^3^ cells per well and incubated at 37°C in a humidified atmosphere containing 5% CO_2_. After 24 h, the cells were treated with different drugs, and cell proliferation was assessed using the CCK‐8 assay kit (Beyotime, C0038) on days 0, 2, 4, and 6.

### 4.4 Cell Apoptosis and Cycle Detection

The Annexin V Alexa Fluor 647/PI Apoptosis Assay Kit (FXP018‐100, 4A Biotech, China) was used to detect apoptosis. Briefly, after treating the cells with different concentrations of the drug for 72 h, the cells were collected, washed with ice‐cold phosphate‐buffered saline (PBS), and resuspended in 100 µL of binding buffer. The cells were then incubated with 5 µL of Annexin V Alexa Fluor 647 solution at room temperature for 5 min. Following this, the cells were stained with 10 µL of propidium iodide (20 µg/mL) and 400 µL of PBS. The stained cells were immediately analyzed by bivariate flow cytometry using a FACSCanto II (BD Accuri C6 FlowCytometer). The drug‐treated cells were collected and washed once with PBS, then fixed with pre‐cold 75% ethanol at −20°C overnight. After the fixation, cells were spun down and washed with PBS, then cells were incubated with the Propidium Iodide (PI) staining buffer containing 0.1% NP‐40, 10ug/ml RNase (Takara, 2158), 1:500 PI (Invitrogen, P3566) in PBS for 30 mins in the dark. Flow cytometry (BD Accuri C6 FlowCytometer) was used to detect the PI signal using the PE‐A channel. The cell cycle was analyzed by the Flowjo software.

### 4.5 Antibacterial Property Evaluation

The antibacterial activity of MTX‐Zn was evaluated based on S.aureus (Staphylococcus aureus ATCC6538) and E.coli (E.coli DH5α strain). They were both obtained from Guangzhou Qiyun Biotechnology Co., Ltd (Guangzhou, China). The MTX‐Zn were put into the test tube containing 4 mL cultured E.coli suspension (1×10^4^ CFUs/mL) and S.aureus suspension (1 × 10^4^ CFUs/mL). After incubation at 37°C and 170 rpm for 12 h, 0.5 mL suspension was extracted from the test tube, and the diluents of E.coli (10^−6^) and S.aureus (10^−5^) were prepared with broth medium. 50 µL diluent was smeared on agar medium and cultured overnight at 37°C. Bacterial colony counting was performed for each sample. Moreover, the bacteriostatic effects of MTX and ZnCl_2_ were evaluated following the same method as mentioned above. Next, drop the bacterial suspension onto a copper grid, stain with negative staining, and perform transmission electron microscopy (TEM) analysis. Additionally, fix the bacterial suspension overnight at 4°C with 2.5% glutaraldehyde, then dehydrate using a gradient of 30%, 50%, 70%, 85%, 95%, and 100% ethanol. After transferring the sample onto a silicon wafer to dry, observe the bacterial morphology using scanning electron microscopy (SEM). All samples were coated with platinum before scanning to improve electrical conductivity.

### 4.6 Synthesis and Characterization of the MTX–Zn/Difelikefalin Dual‐Layer Microneedle (MZ/D‐DL‐MN)

A GelMA solution containing MTX‐Zn was added to the PDMS microneedle mold. After degassing under vacuum, the excess GelMA was scraped off, ensuring it filled the tip of the microneedle mold. The mold was then dried overnight at 40°C, during which the GelMA liquid level decreased. Next, a PVA solution containing DFK was added to the mold, degassed, and dried. Subsequently, another layer of PVA solution was added to form the base of the microneedles. The structure was then freeze‐dried to remove any remaining moisture from the microneedles. Scanning electron microscopy (SEM) with an acceleration voltage of 10 kV was used to observe the micromorphology of the MTX‐Zn/DFK Dual‐Layer Microneedle (MZ/D‐DL‐MN) in order to reveal its structural characteristics.

### 4.7 Mechanical Strength Test and Separation Behavior of Microneedle Tips

The mechanical strength of the microneedle (MN) was tested using a compression testing machine (ZQ‐990B, Zhiqu Precision Instrument, China) with descending force. The microneedle was securely positioned on the tensile meter, and the pressure sensor applied a gradual force at a constant speed of 0.1 mm/min. A sufficient amount of physiological saline was added to the culture dish containing the fixed MZ/D‐DL‐MN, ensuring that the liquid level surpassed the height of the microneedle. The degradation of the microneedle tips' base was then observed and recorded using a stereo microscope.

### 4.8 In Vitro Release Behavior of MTX‐Zn and Difelikefalin from MZ/D‐DL‐MN

MZ/D‐DL‐MN was placed in 30 mL of physiological saline and incubated at 37°C on a shaker for continuous oscillation. At specified time intervals, 1 mL of the solution was withdrawn and replaced with 1 mL of fresh physiological saline to maintain a constant volume. The concentrations of MTX‐Zn and Difelikefalin in the solution were analyzed by high‐performance liquid chromatography (HPLC) to determine the release rate.

### 4.9 Skin Insertion Ability of MN

The mice were depilated and allowed to acclimate for one day to restore the cuticle layer. Subsequently, gentle thumb pressure was applied vertically to the skin with the microneedle for 10 s. After removing the microneedle, the mouse skin was carefully excised and prepared into frozen sections with a plane perpendicular to the skin tissue for microscopic observation. Using the same method, after the microneedle was inserted into the mouse skin, the skin tissue along with the microneedle was embedded in Optimum Cutting Temperature (OCT) compound, and serial multi‐layer frozen sections were prepared with a plane parallel to the skin surface. Imaging was performed using a confocal fluorescence microscope.

### 4.10 Cell Migration Assay

Schwann cells were seeded in six‐well plates. Upon reaching approximately 80–90% confluence, a vertical scratch was made in the cell monolayer of each well using a 200 µL pipette tip. The wells were then washed with medium to remove detached cells. The experimental group was treated with 20 µm difelikefalin. Microscopic images were captured at 0 h, 12 h, 24 h, 36 h, and 48 h to monitor cell migration. To further assess the migration ability of Schwann cells, 1 × 104 cells/well were plated in the upper compartment of a transwell chamber (pore size 8 µm). The upper chamber contained serum‐free medium, while the lower chamber contained 600 µL of complete medium with 10% fetal bovine serum. Both the upper and lower chambers were supplemented with the same concentration of difelikefalin. After 12 h, the cells remaining in the upper chamber were gently wiped off with a cotton swab. The transwell chamber was then fixed with 4% formaldehyde for 15 min, followed by staining with 0.1% crystal violet for 20 min. The chambers were washed three times with PBS, and images were captured and recorded. The number of cells that migrated to the underside of the transwell was quantified using Image J.

### 4.11 In Vivo Treatment of Psoriasis Using MZ/D‐DL‐MN

C57BL/6 mice (8–10 weeks old, weighing 20–25 g) were depilated under anesthesia. The mice were allowed to recover and feed normally for 24 h to restore the stratum corneum. Imiquimod (IMQ) cream (62.5 mg) was then applied to the depilated area on the back of the mice for seven consecutive days. The experimental groups include: Control group, Blank MNs, DFK MNs, MTX‐Zn MNs, MTX‐Zn/DFK MNs. During the 7‐day treatment period, the psoriasis severity index score and body weight of the mice were recorded. The Psoriasis Area Severity Index (PASI) score was determined based on erythema, psoriasis, and sclerosis: 0 (asymptomatic), 1 (mild), 2 (moderate), 3 (severe), or 4 (very severe). On day 7, the mice were euthanized, and skin tissue and spleen were collected. The spleen was imaged with a cell phone camera, and its weight was recorded. Skin tissue was fixed with 4% paraformaldehyde, and stained with HE for microscopic examination.

### 4.12 Flow Cytometry Assays

Spleens were aseptically harvested from euthanized mice and immediately placed in cold PBS. Each spleen was transferred to a 70 µm cell strainer positioned over a sterile 6‐well plate and gently dissociated using the plunger end of a syringe to obtain a single‐cell suspension. The resulting cell suspension was collected and carefully layered onto lymphocyte separation medium according to the manufacturer's instructions. Samples were centrifuged at 800 × *g* for 20 min at room temperature without brake. After centrifugation, the mononuclear cell layer at the interface was carefully aspirated, transferred to a new tube, and washed twice with PBS (400 × *g*, 5 min). Then the cells were stained by Anti‐CD4, Anti‐CD8, Anti‐Foxp3, Anti‐LY6G, Anti‐CD11B, Anti‐F4/80, Anti‐IL17A, anti‐IFN‐γ to analyze the ratio changes of immune cells. The antibodies were all obtained from BioLegend.

### 4.13 Multiplex Immunofluorescence Staining (mIHC) of Mouse Skin Tissue

Multiplex immunofluorescence staining was performed using the PANO 7‐plex IHC kit (Panovue, Beijing, China). Briefly, slides were incubated at 65°C for 2 h, deparaffinized with xylene and a graded ethanol series (100%, 95%, 70%, 50%), and fixed in 10% neutral buffered formalin for 30 min. Antigen retrieval was performed using microwave treatment with EDTA buffer (pH 9.0, ZSGB‐Bio, Beijing, China), followed by blocking. Tyramide signal amplification (TSA) was performed after sequential incubation with primary antibodies: S100B (1:200, 90393, CST) and PGP9.5 (1:200, 14730‐1‐AP, Proteintech). Sections were then treated with HRP‐conjugated secondary antibodies (anti‐rabbit HRP, 1:10,000, HS101; TransGen Biotech) and HRP‐conjugated streptavidin (Panovue, Beijing, China). Biotinylated secondary antibodies were used for streptavidin‐linked alkaline phosphatase‐dependent chromogen reactions and fluorophore detection. Imaging was performed using a confocal fluorescence microscope.

### 4.14 Transcriptome Sequencing Analysis

Gene expression analysis of the collected difelikefalin‐treated Schwann cells was performed using the RNA sequencing platform (BerryGenomics Co., Ltd.). RNA was extracted using the TRIzol method, and cDNA library construction, purification, and RNA sequencing were carried out according to standard protocols. The RNA quality was assessed using an Agilent 2100 Bioanalyzer (RIN>9). Sequencing reads were aligned to the mouse genome using HISAT2, retaining only the uniquely mapped reads. Differential expression analysis was performed using DESeq2. Bioinformatics analysis, including Hallmark Gene Set Enrichment Analysis and KEGG Gene Set Enrichment Analysis, was conducted using R software (version 4.1.3) with a threshold of p‐adjusted <0.05 and fold change >2.

### 4.15 Real‐Time PCR

Total RNA was extracted using the Trizol reagent according to the manufacturer's instructions. Reverse transcription (RT) was performed using the EvoM‐MLV Reverse Transcription Kit, following the provided protocol. The qPCR reaction was prepared using the SYBR Green Premix Pro TaqHS Kit, Trizol, and qPCR was then conducted using the QuantStudio 12K Flex Real‐Time PCR System. The primers are listed in Table 2. Cells were lysed using RIPA buffer (Cell Signaling Technology, USA) for protein extraction. Protein concentration was determined using the BCA working solution (ThermoFisher). The denatured proteins were separated by SDS‐PAGE and transferred onto PVDF membranes. The membranes were incubated overnight at 4°C with primary antibodies (1:1000), including ZEB1, Vimentin, Snai1, GAPDH, Twist1. The primary antibodies were purchased from Proteintech, with the catalog numbers 21544‐1‐AP, 10366‐1‐AP, 13099‐1‐AP, 60004‐1‐Ig, and 25465‐1‐AP. After incubation with HRP‐conjugated secondary antibodies, the membranes were treated with ECL reagent. Protein bands were visualized using a Chemiluminescence Imaging System.

**TABLE 2:** Primers used for RT‐qPCR.

Gene name	Sequences (5′‐3′)
ZEB1	R: CATTCTGGTCCTCCACAGTGGA; F: ATTCAGCTACTGTGAGCCCTGC
Vimentin	R: AGCAGTGAGGTCAGGCTTGGAA; F: CGGAAAGTGGAATCCTTGCAGG
Snai1	R: CTTCACATCCGAGTGGGTTTGG; F: TGTCTGCACGACCTGTGGAAAG
Twist1	R: AGACGGAGAAGGCGTAGCTGAG; F: GATTCAGACCCTCAAACTGGCG
GAPDH	R:ATGCCAGTGAGCTTCCCGTTCAG; F: CATCACTGCCACCCAGAAGACTG

### 4.16 Statistical Analysis

The sample size for each experiment was determined according to our previous experiences. The data were expressed as mean±standard deviation (SD), as indicated in the figure legends. Student's *t*‐test and one‐way analysis of variance (ANOVA) were used in this study. Differences with *p* < 0.05 were considered statistically significant.

## Conflicts of Interest

The authors declare no conflicts of interest.

## Supporting information




**Supporting File**: smll73958‐sup‐0001‐FigureS1.pdf.

## Data Availability

The data that support the findings of this study are available from the corresponding author upon reasonable request.
