# Supplementary material for: Innovative Microneedle Patches for Psoriasis Treatment: A Dual Approach With Methotrexate‐Zinc and Difelikefalin for Enhanced Therapeutic Outcomes
Source: Small. 2026 May 30;22(36):e73958. doi: 10.1002/smll.73958 (PMC13306916; doi:10.1002/smll.73958)
Supplement: Supplementary file 1 — Supporting File: smll73958‐sup‐0001‐FigureS1.pdf. [file SMLL-22-e73958-s001.pdf]

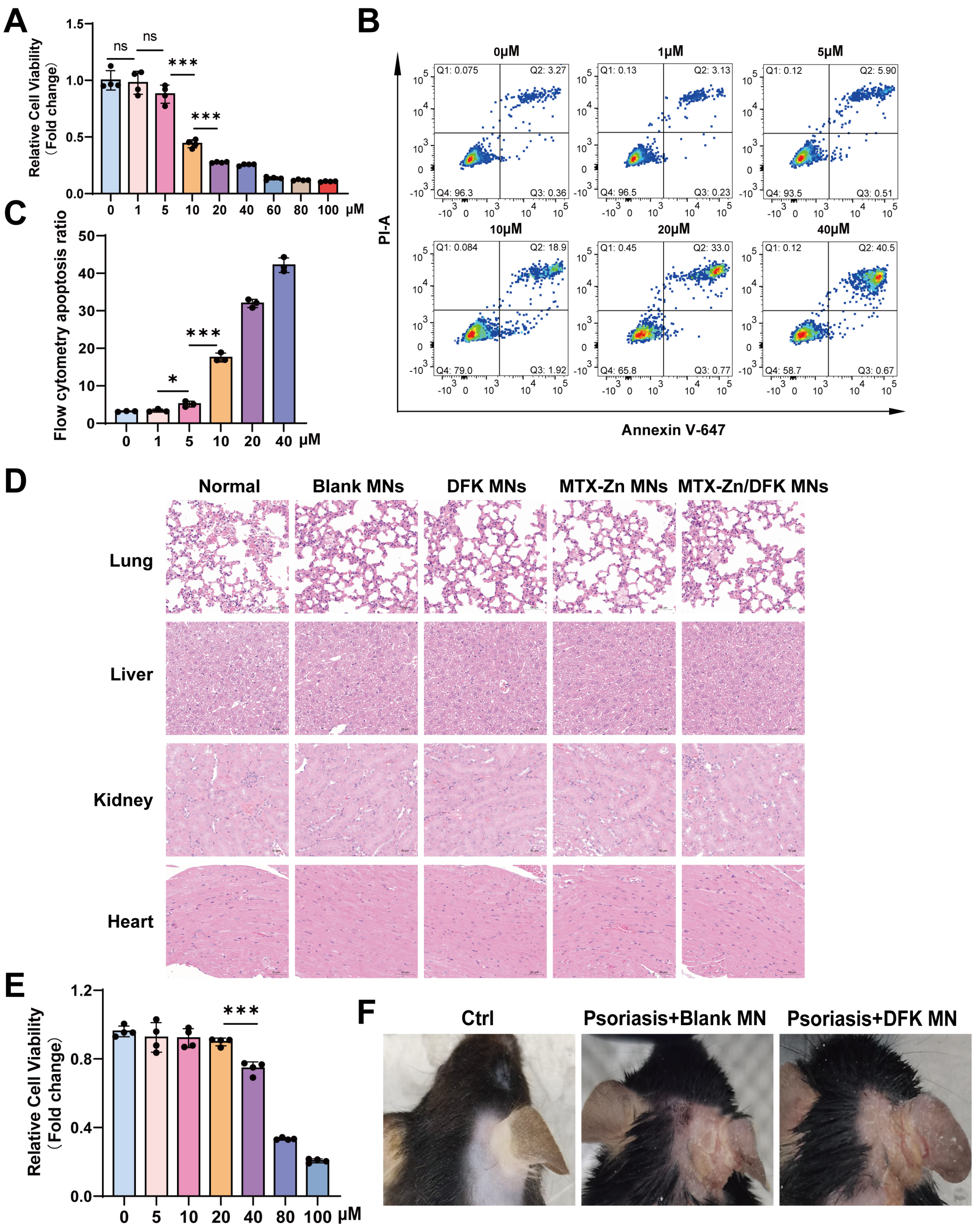

Fig. S1. Effects of methotrexate (MTX) on the viability and apoptosis of keratinocytes.
(A) Cell viability of keratinocytes treated with increasing concentrations of methotrexate (0-100 μM) for 72 h, measured using the CCK-8 assay. (B) Representative flow cytometry plots showing apoptosis of keratinocytes after 72 h of methotrexate treatment. (C) Quantification of apoptosis percentages corresponding to the flow cytometry data in panel B. (D) HE staining of lung, liver, kidney, and heart tissues from all treatment groups showing no significant histopathological abnormalities. (E) Schwann cells were treated with difelikefalin for 72 hours, and cell viability was evaluated using the CCK-8 assay. (F) After the establishment of the ear psoriasis-like model, mice were treated with different microneedles, and their scratching behavior was monitored. Data are presented as mean ± SD. ns, not significant; $*P < 0.05$, $***P < 0.001$.
